# Molecular detection of latent feline leukemia virus infection using pol gene polymerase chain reaction in an enriched clinical cohort of Thai cats

**DOI:** 10.14202/vetworld.2026.4115-4132

**Published:** 2026-09-18

**Authors:** Kanthapan Ubolrat, Amon Praduptong, Sudarat Chaichalerm, Chaiwat Boonkaewwan, Phatthadon Thamrongthanaset, Jatuporn Rattanasrisomporn, Atchaya Rapichai

**Affiliations:** 1 Faculty of Veterinary Medicine, Phetchaburi Rajabhat University, Phetchaburi 76120, Thailand.; 2 Department of Public Health, Faculty of Nursing and Allied Health Science, Phetchaburi Rajabhat University, Phetchaburi 76120, Thailand.; 3 Department of Companion Animal Clinical Sciences, Faculty of Veterinary Medicine, Kasetsart University, Bangkok 10900, Thailand.

**Keywords:** antigen detection, diagnostic accuracy, feline leukemia virus, latent infection, molecular diagnosis, polymerase chain reaction, proviral DNA, Thailand

## Abstract

**Background and Aim:**

Feline leukemia virus (FeLV) infection remains an important cause of morbidity and mortality in domestic cats. Conventional p27 antigen assays are widely used for screening but may fail to detect proviral DNA in cats without detectable antigenemia. This study aimed to evaluate a polymerase chain reaction (PCR) assay targeting the conserved FeLV *pol* gene for molecular detection of FeLV and to compare its diagnostic performance with a commercial p27 antigen rapid immunoassay in an enriched clinical cohort of Thai cats.

**Materials and Methods:**

A total of 160 residual ethylenediaminetetraacetic acid-blood specimens were obtained from an enriched referral cohort of cats presented to Kasetsart University Veterinary Teaching Hospital for FeLV screening or blood-donor qualification. Proviral DNA was analyzed by conventional PCR targeting an approximately 791-bp fragment of the FeLV *pol* gene, and results were compared with those of the commercial p27 antigen assay. PCR products from discordant specimens were subjected to Sanger sequencing and sequence comparison with reference FeLV *pol* sequences. Diagnostic sensitivity, specificity, positive predictive value (PPV), negative predictive value (NPV), and overall accuracy were determined.

**Results:**

FeLV proviral DNA was detected by *pol* gene PCR in 137/160 (85.6%) specimens, whereas 94/160 (58.8%) were positive by the p27 antigen assay. All 94 antigen-positive specimens were PCR-positive, while 43/160 (26.9%) were PCR-positive but antigen-negative. Using PCR as the reference standard, the antigen assay showed 68.61% sensitivity (95% confidence interval: 60.13–76.27%), 100% specificity, 100% PPV, 34.8% NPV, and 73.13% overall accuracy. Sanger sequencing of discordant PCR-positive/antigen-negative specimens demonstrated 98% nucleotide identity with reference FeLV *pol* sequences, supporting the presence of FeLV proviral DNA in these antigen-negative specimens.

**Conclusion:**

The *pol* gene PCR detected substantially more FeLV-positive specimens than p27 antigen testing in this enriched clinical cohort, particularly among antigen-negative cats harboring detectable proviral DNA. Incorporating molecular testing as a complementary method may improve detection in high-risk cats, blood-donor screening, and cases with discordant or clinically suspected FeLV infection. Because the cohort was deliberately enriched, the observed positivity rates should not be extrapolated to the general Thai cat population.

## INTRODUCTION

Feline leukemia virus (FeLV), a gammaretrovirus belonging to the family *Retroviridae*, is one of the most important retroviral pathogens affecting domestic cats worldwide. The FeLV genome consists of a linear, single-stranded RNA molecule of approximately 8.3 kb. The *gag* and *pol* genes occur within the same translational reading frame and encode structural and enzymatic proteins essential for viral replication and pathogenesis, respectively. In particular, the *pol* gene encodes the nonstructural enzymatic proteins p14 protease, p80 reverse transcriptase, and p46 integrase, which are required for proviral DNA synthesis and chromosomal integration. Because these enzymatic functions are essential to the viral life cycle and the *pol* nucleotide sequence is highly conserved among diverse FeLV isolates, the *pol* gene represents a suitable molecular target for detecting proviral DNA, including in cats without detectable circulating viral proteins [1, 2].

Following exposure to FeLV, the interaction between viral replication and the host immune response can result in abortive, regressive, or progressive infection. In abortive infection, an effective host immune response eliminates the virus before proviral infection is established. Progressive infection is characterized by persistent viremia and sustained viral replication, resulting in circulating p27 capsid antigen that can generally be detected using conventional antigen-based screening assays. In contrast, regressive infection occurs when viral replication is suppressed after an initial period of infection, while proviral DNA remains integrated within the host genome. Consequently, cats with regressive infection may lack detectable circulating p27 antigen despite harboring FeLV proviral DNA. Such cats can remain clinically asymptomatic for prolonged periods, and viral reactivation may occur under immunosuppression or concurrent disease [3–7].

In Thailand, FeLV surveillance and clinical diagnosis commonly rely on rapid p27 antigen immunochro-matographic assays because of their convenience, rapid turnaround time, and suitability for point-of-care use. However, relying on antigen detection alone may fail to identify cats with proviral DNA but without detectable antigenemia. Recent epidemiological evidence from Thailand has shown regressive FeLV infection among domestic cats, emphasizing that antigen-based screening alone may underestimate the burden of FeLV infection [8]. This diagnostic limitation may be particularly relevant in environments involving close contact among cats, including animal shelters, multi-cat households, and breeding catteries, where accurate determination of infection status is important for disease-control decisions [6, 9–11].

Polymerase chain reaction (PCR)-based detection of conserved FeLV genomic regions provides a complementary approach for identifying proviral DNA when circulating viral antigen is absent. Because PCR detects viral nucleic acid rather than viral protein expression, it can identify integrated provirus in cats with suppressed viral replication [12, 13]. The relatively high sequence conservation of the *pol* gene among FeLV strains further supports its use as a molecular target and may reduce the potential effect of sequence variation on primer recognition [2, 14–17]. Therefore, combining antigen-based screening with molecular detection may provide a more complete assessment of FeLV infection status than either approach alone.

Despite the recognized value of molecular detection, information on antigen–provirus discordance and regressive FeLV infection in Thai cats remains limited. Previous regional investigations have relied substantially on p27 antigen detection, which cannot independently establish whether antigen-negative cats harbor integrated FeLV proviral DNA. Consequently, the magnitude of potentially undetected infection within clinically relevant Thai cat populations remains insufficiently characterized. Moreover, direct evaluation of commercially available p27 antigen testing against *pol* gene PCR, together with sequence confirmation of discordant PCR-positive/antigen-negative specimens, remains limited in Thailand [15, 18–21]. This gap is particularly relevant to clinical screening and blood-donor assessment, in which failure to identify provirus-positive cats could influence interpretation of FeLV infection status.

Another methodological gap concerns confirming discordant molecular findings. Detection of a PCR amplicon alone establishes the presence of a product of the expected size but does not independently verify its sequence identity. Sequence confirmation of PCR-positive/antigen-negative specimens can therefore strengthen evidence that discordance represents detection of FeLV proviral DNA rather than nonspecific amplification. Evaluating this discordance within an enriched clinical cohort can provide clinically relevant information regarding the additional detection achieved by molecular testing. However, because such a cohort is not population-representative, positivity estimates must be distinguished from estimates of FeLV prevalence in the general Thai cat population.

The present study aimed to evaluate a PCR assay targeting the conserved FeLV *pol* gene for detection of proviral DNA and to compare its diagnostic performance with a commercial p27 antigen rapid immunoassay in an enriched clinical cohort of Thai cats. Specifically, the study sought to determine the frequency and pattern of agreement and discordance between *pol* gene PCR and p27 antigen results, characterize PCR-positive/antigen-negative specimens, and assess the diagnostic performance of the antigen assay using *pol* gene PCR as the molecular reference method. In addition, selected discordant PCR products were subjected to Sanger sequencing to confirm their identity through comparison with reference FeLV *pol* sequences. Through this combined molecular and antigen-based approach, the study was intended to provide evidence supporting more comprehensive interpretation of FeLV screening results and the potential use of molecular confirmation in clinically relevant cats with negative or discordant antigen findings.

## MATERIALS AND METHODS

### Ethical approval

The study was conducted in accordance with the National Research Council of Thailand’s Ethical Principles and Guidelines for the Use of Animals for Scientific Purposes. The study protocol was reviewed and approved by the Institutional Animal Care and Use Committee (IACUC) of Kasetsart University, Bangkok, Thailand (Approval No. ACKU69-VET-014; approved January 12, 2026).

All blood samples analyzed in this study were archived residual specimens originally collected as part of routine clinical diagnostic procedures, including FeLV screening and blood-donor qualification, and no additional blood collection or animal manipulation was performed specifically for this study. At the time of hospitalization, cat owners provided consent through the standard Kasetsart University Veterinary Teaching Hospital admission form, which includes a specific provision permitting the use of residual biological specimens for future ethically approved research, subject to anonymization of patient data. Only residual specimens from cats whose owners had provided this consent were eligible for inclusion in the study.

Before analysis, all patient information was fully anonymized, and the research database retained no personally identifiable information, including owner names, addresses, or patient registration numbers. Because the study used archived residual clinical specimens, involved no additional intervention or animal manipulation, and used anonymized data, the IACUC classified it as non-interventional and waived the requirement for study-specific prospective re-consent based on the broad consent previously obtained from owners at hospital admission.

### Study period and location

The study was conducted from January to March 2025 using residual feline ethylenediaminetetraacetic acid (EDTA)-blood specimens archived at the Blood Bank Unit of Kasetsart University Veterinary Teaching Hospital, Thailand. The specimens originated from client-owned cats presented for FeLV screening or blood-donor qualification during the defined 3-month study period.

### Study design

This cross-sectional diagnostic accuracy study used a convenience sample from an enriched hospital cohort to compare FeLV *pol* gene PCR with a commercial p27 antigen rapid immunoassay (Zoetis Inc., Parsippany, NJ, USA) in a Thai clinical setting. A total of 160 residual EDTA-blood specimens were retrieved from the Blood Bank Unit archives of cats presented for FeLV screening or blood-donor qualification. The cohort was intentionally enriched by preferentially selecting residual specimens from cats with a documented history of FeLV testing (including previous positive, discordant, or high-risk clinical cases) to increase the expected frequency of proviral DNA detection and antigen–provirus discordance relative to the general feline population. Because this was an enriched clinical cohort rather than a population-based sample, the proportion of PCR-positive specimens was not considered representative of FeLV prevalence in the general Thai cat population. The overall experimental workflow, including sample processing, molecular detection of FeLV proviral DNA targeting the *pol* gene, comparison with p27 antigen testing, and sequence confirmation of discordant specimens, is summarized in Figure 1.

### Sample size calculation

The target sample size was determined based on the minimum requirements for diagnostic accuracy studies comparing two binary tests. Using the formula for estimating sensitivity with specified precision, we assumed an expected PCR sensitivity of 90%, based on the reported performance of FeLV *pol* gene PCR [22], with a desired precision of ±10% at the 95% confidence level. This calculation indicated that at least 87 true-positive specimens were required. Given the anticipated higher frequency of FeLV proviral DNA in the enriched study cohort, a target enrollment of 140–160 specimens was established to ensure an adequate number of positive cases. The final sample comprised 160 specimens, including 137 PCR-positive cases, thereby exceeding the prespecified target. When the p27 antigen assay was evaluated against *pol* gene PCR as the reference method, this sample size provided an approximate 95% confidence interval (CI) precision of ±7.6% for sensitivity.

For specificity estimation, the 23 PCR-negative specimens observed in the final dataset provided a 95% CI of 85.2%–100% (assuming an expected specificity of ≥95%). This level of precision met the minimum requirements for diagnostic test validation described in the OIE Terrestrial Manual [23].

### Inclusion and exclusion criteria

Residual specimens were eligible for inclusion when they met the following criteria: EDTA-anticoagulated whole blood collected from client-owned cats presented to Kasetsart University Veterinary Teaching Hospital for FeLV screening or blood-donor qualification; a minimum residual volume of 200 µL after completion of clinically requested diagnostic testing; availability of a concurrent p27 antigen test result in the clinical records; collection between January and March 2025; and storage at −20°C for no longer than 90 days before PCR analysis.

**Figure 1:**
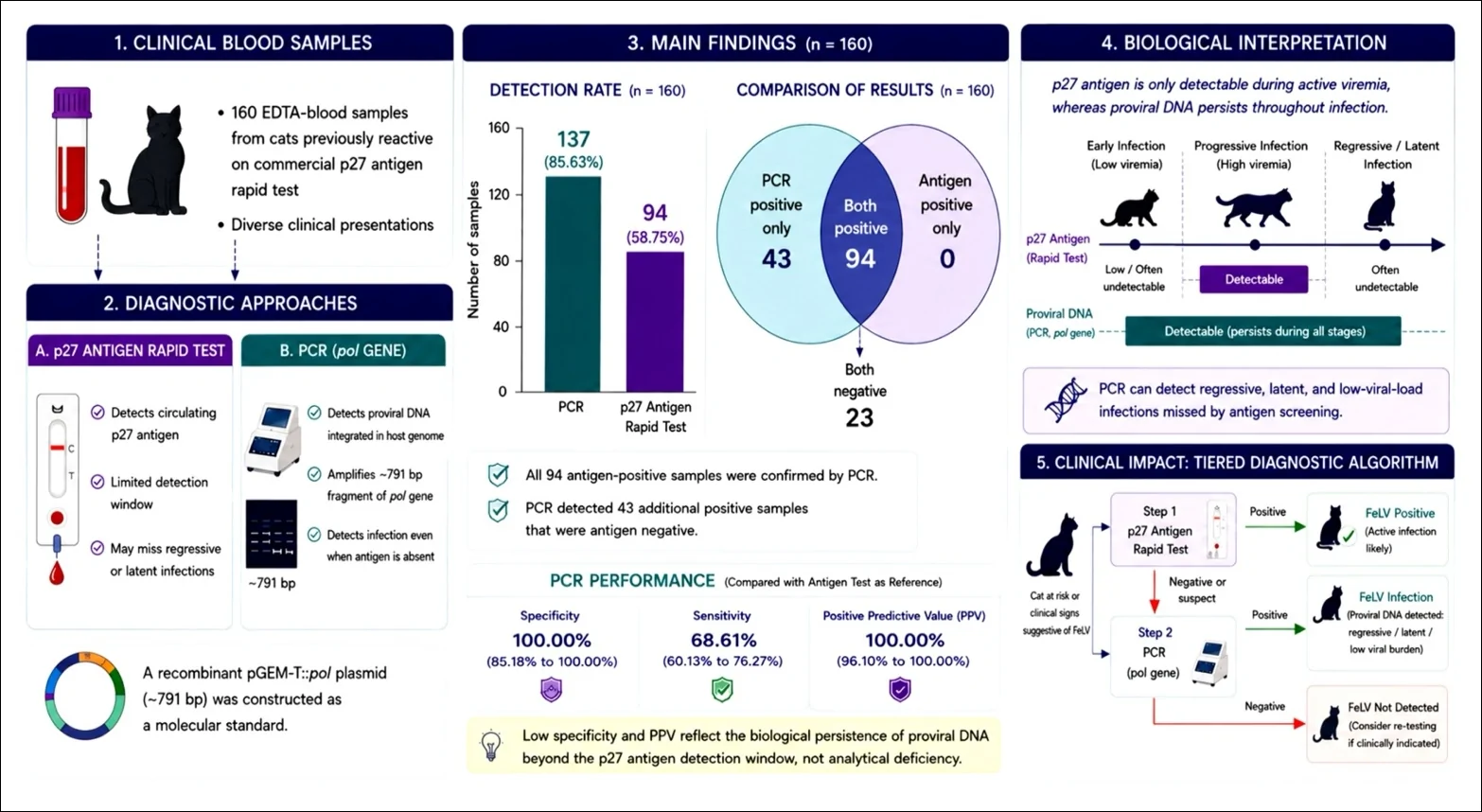
The *pol* gene polymerase chain reaction detects FeLV proviral DNA in at-risk Thai cats missed by p27 antigen testing, revealing potentially silent viral reservoirs within an enriched clinical population.

Specimens were excluded if they showed visible hemolysis, lipemia, or clotting that could interfere with DNA extraction; originated from cats receiving documented concurrent immunosuppressive treatment, including glucocorticoids or cyclosporine, at the time of collection; had a residual volume <200 µL; lacked complete signalment data for breed, age, or sex; or represented duplicate submissions from the same cat. When duplicate submissions were identified, only the most recent specimen was retained.

### Sampling method

A convenience sampling strategy was used. Potentially eligible specimens were identified retrospectively from the Blood Bank Unit electronic database, and all archived residual specimens satisfying the predefined eligibility criteria and available during January–March 2025 were retrieved for testing. The specimens did not constitute a consecutive clinical series because their availability depended on clinical submission volumes and the amount of residual blood remaining after routine diagnostic procedures. No additional selection or stratification was applied beyond the predefined inclusion and exclusion criteria.

### DNA extraction from feline blood samples

Proviral DNA was extracted from EDTA-anticoagulated whole blood using the E.Z.N.A. Universal Pathogen Kit (Omega Bio-Tek, Norcross, GA, USA) according to the manufacturer's instructions. Because the FeLV replication cycle involves reverse-transcription of viral genomic RNA into DNA followed by integration into the host genome, detection of proviral DNA in peripheral blood leukocytes provides molecular evidence of FeLV infection [14].

Extracted DNA was quantified using a NanoDrop 2000 spectrophotometer (Thermo Fisher Scientific, Waltham, MA, USA) at 260 nm, and purity was evaluated using the A260/A280 and A260/A230 ratios. Samples with A260/A280 ratios of 1.7–2.0 and A260/A230 ratios ≥1.5 were considered acceptable for PCR analysis. Across the 160 specimens, the mean DNA concentration was 45.2 ± 18.7 ng/µL (range, 12.3–112.6 ng/µL), with mean A260/A280 and A260/A230 ratios of 1.85 ± 0.06 and 2.08 ± 0.12, respectively. DNA integrity was additionally evaluated in 20 randomly selected specimens by electrophoresis on a 0.8% agarose gel. All extracted DNA was eluted in 50 µL of preheated (70°C) elution buffer, stored at −20°C in low-binding microcentrifuge tubes, and subjected to no more than two freeze–thaw cycles before PCR analysis. All 160 specimens met the predefined purity criteria and were processed without re-extraction or dilution.

### Construction of the standard plasmid harboring the FeLV *pol* gene

Total RNA corresponding to FeLV was extracted from the Leukocell®2 FeLV vaccine (Zoetis Inc., Parsippany, NJ, USA) using the E.Z.N.A. Viral RNA Kit (Omega Bio-Tek, Norcross, GA, USA) according to the manufacturer's protocol. Purified RNA was subjected to reverse-transcription-PCR (RT-PCR) using the Deoxy⁺ OneStep RT-PCR Kit (Yeastern Biotech, New Taipei City, Taiwan). The *pol*-F (5′-CYAMCCRTTATTRGGDAGAGA-3′) and *pol*-R (5′-CCAGCAAGAGGTCATCTACA-3′) primers were adapted from Ramírez *et al.* [24].

Each 25-µL RT-PCR reaction contained 12.5 µL of 2× premix, 1.38 µL of each primer (10 µM), 7.74 µL of RNase-free water, and 2 µL of RNA template. Reverse-transcription was performed at 42°C for 60 min, followed by enzyme inactivation at 70°C for 7 min. Amplification consisted of 45 cycles of denaturation at 94°C for 1 min, annealing at 55°C for 45 s, and extension at 72°C for 50 s, followed by a final extension at 72°C for 10 min. The expected approximately 791-bp amplicon was separated on a 1.5% agarose gel, excised, and purified using the GeneAll® Expin™ Combo GP Kit (GeneAll Research Institute, Seoul, Korea).

The purified amplicon was ligated into the pGEM-T vector (Promega, Madison, WI, USA) using T4 DNA ligase according to the manufacturer's instructions to generate pGEM-T::*pol*. The ligation mixture was transformed into chemically competent *Escherichia coli* DH5α cells (Subcloning Efficiency™ DH5α™, Thermo Fisher Scientific, Waltham, MA, USA) by heat shock at 42°C for 45 s, followed immediately by incubation on ice for 2 min. Transformed cells were recovered in 1 mL of Super Optimal Broth (Thermo Fisher Scientific) with catabolite repression medium at 37°C with agitation at 200 rpm for 1 h.

Blue–white screening was performed on Luria–Bertani medium (LB) agar (Merck KGaA, Darmstadt, Germany) supplemented with 100 µg/mL ampicillin (Merck KGaA), 0.5 mM isopropyl β-D-1-thiogalactopyranoside (Merck KGaA), and 40 µg/mL 5-bromo-4-chloro-3-indolyl-β-D-galactopyranoside (Merck KGaA). White colonies were selected and screened by colony PCR using M13F and M13R universal primers. Plasmid DNA from PCR-positive colonies was purified and quantified using a NanoDrop 2000 spectrophotometer (Thermo Fisher Scientific). The resulting recombinant plasmid was subsequently used as a reference standard and positive control for PCR.

### Sequence confirmation of the recombinant plasmid

Positive recombinant clones harboring the FeLV *pol* gene were cultured overnight in 5 mL of LB broth (Merck KGaA) containing 100 µg/mL ampicillin (Merck KGaA) at 37°C with agitation at 200 rpm. Cells were harvested by centrifugation at 8,000 × *g* for 3 min, and recombinant plasmid DNA was purified using the GeneAll® Expin™ Combo GP Kit (GeneAll Research Institute). DNA concentration and purity were evaluated using a NanoDrop 2000 spectrophotometer (Thermo Fisher Scientific).

The purified recombinant plasmid was subjected to bidirectional Sanger sequencing using M13F (5′-GTAAAACGACGGCCAG-3′) and M13R (5′-CAGGAAACAGCTATGAC-3′) universal primers. Sequencing was performed using an ABI 3730xl DNA Analyzer (Applied Biosystems, Foster City, CA, USA) with the BigDye Terminator v3.1 Cycle Sequencing Kit. The resulting consensus sequence was compared with FeLV reference sequences available in GenBank using the Basic Local Alignment Search Tool (BLAST) to verify the identity of the cloned *pol* gene fragment.

### Rapid immunochromatographic detection of FeLV p27 antigen

FeLV p27 antigen results were obtained from the original clinical records of the Blood Bank Unit. Testing was performed by a veterinarian using a commercial point-of-care rapid immunochromatographic assay (Zoetis Inc., Parsippany, NJ, USA). The specific kit name, manufacturer, and lot number were not recorded in the residual specimen archive. Before testing, the assay components and feline blood samples were equilibrated to room temperature for 30 min. Three drops of whole blood were mixed with three drops of conjugate solution containing monoclonal antibodies against FeLV p27 antigen. A visible control line indicated a valid assay, and the appearance of a test line in the FeLV detection window was interpreted as a positive result. All tests were performed and interpreted by a single experienced veterinarian.

### PCR assay for FeLV *pol* gene detection

Clinical specimens were tested for FeLV proviral DNA using the same *pol*-F and *pol*-R primer pair described above. The primer sequences and amplification conditions were adapted from Ramírez *et al.* [24]. Each 25-µL PCR mixture contained 1× DreamTaq Green PCR Master Mix (Thermo Fisher Scientific, Wilmington, NC, USA), 0.6 µM each of the *pol*-F and *pol*-R primers, 8.5 µL of RNase-free water, and 1 µL of DNA extracted from feline blood.

A no-template control containing all PCR components except DNA template was included in every amplification run to monitor contamination. The pGEM-T::*pol* recombinant plasmid at 10⁵ copies served as the positive control, whereas RNase-free water served as the negative amplification control. PCR products were separated by electrophoresis on a 1.5% agarose gel prepared in 1× Tris-acetate-EDTA buffer (pH 8.0), stained with Novel Juice (GeneDireX, Taoyuan City, Taiwan), and visualized using a BluPAD LED Transilluminator (BIO-HELIX, New Taipei City, Taiwan). The expected positive product was approximately 791 bp. No amplification was observed in the no-template controls. Formal analytical limit of detection, cross-reactivity, and intra- or inter-assay reproducibility were not determined in this clinical diagnostic accuracy study.

### Statistical analysis

Concordance between *pol* gene PCR and the p27 antigen immunoassay was summarized using a 2 × 2 contingency table. Sensitivity, specificity, positive predictive value (PPV), and negative predictive value (NPV) of the commercial p27 antigen assay were calculated as percentages using MedCalc’s Diagnostic Test Evaluation Calculator, with *pol* gene PCR designated as the molecular reference method. Analyses were conducted under blinded conditions to minimize interpretation bias.

## RESULTS

### Positive recombinant plasmid construction

RT-PCR amplification of the FeLV *pol* gene from the Leukocell®2 vaccine successfully generated an approximately 791-bp fragment, which was subsequently purified and cloned into the pGEM-T vector. Following transformation into *Escherichia coli* DH5α cells, recombinant clones containing the target insert were successfully obtained and verified. Sanger sequencing provided complete sequence coverage of the cloned fragment, as shown in Figure S1. BLAST analysis demonstrated 98% nucleotide identity with the Feline leukemia virus isolate FeLV_US_x1948_Pco2004 (GenBank accession no. MF681672). The high sequence identity confirmed that the recombinant construct contained the intended FeLV *pol* gene fragment and supported the use of the pGEM-T::*pol* plasmid as a molecular reference standard and positive control for subsequent PCR analyses.

### Study population

A total of 160 residual EDTA-anticoagulated blood specimens were retrieved from the Blood Bank Unit archives of Kasetsart University Veterinary Teaching Hospital, Thailand. These archived specimens represented an enriched referral cohort of cats presented for FeLV screening or blood-donor qualification. The cohort was intentionally enriched by preferentially selecting residual specimens from cats with a documented history of FeLV testing (including previous positive, discordant, or high-risk clinical cases) to increase the expected frequency of proviral DNA and antigen–provirus discordance relative to the general feline population. The demographic characteristics of the 160 cats are summarized in Table 1.

**Table 1:** Demographic characteristics of the 160 cats from which residual blood specimens were obtained.

**Variable**	**Category**	**n**	**%**
Sex	Male	92	57.5
	Female	68	42.5
Breed	Domestic Shorthair	112	70.0
	Persian	17	10.6
	Scottish Fold	10	6.3
	British Shorthair	9	5.6
	American Shorthair	6	3.8
	Siamese	3	1.9
	Maine Coon	3	1.9
Age group	<1 year	29	18.1
	1–3 years	47	29.4
	4–6 years	36	22.5
	7–10 years	14	8.8
	>10 years	34	21.3

The study population comprised 92 male cats (57.5%) and 68 female cats (42.5%), indicating a modest predominance of males. The cats represented a broad age range: 29 (18.1%) were <1 year old, 47 (29.4%) were 1–3 years old, 36 (22.5%) were 4–6 years old, 14 (8.8%) were 7–10 years old, and 34 (21.3%) were >10 years old. Overall, 83/160 cats (51.9%) were 1–6 years old, representing the largest age segment of the study population. Juvenile cats aged <1 year and older cats aged >10 years together accounted for 63/160 (39.4%) of the cohort. Thus, the study included cats across juvenile, adult, and senior age categories rather than being restricted to a narrow age group.

Domestic Shorthair was the predominant breed, accounting for 112/160 cats (70.0%). Pedigree breeds comprised the remaining 48 cats (30.0%) and included Persian (17/160; 10.6%), Scottish Fold (10/160; 6.3%), British Shorthair (9/160; 5.6%), American Shorthair (6/160; 3.8%), Siamese (3/160; 1.9%), and Maine Coon (3/160; 1.9%) cats. The predominance of Domestic Shorthair cats reflected the composition of the archived clinical specimens available from the veterinary teaching hospital, while the inclusion of several pedigree breeds represented cats from diverse breed backgrounds.

Overall, the cohort was predominantly male Domestic Shorthair cats and included a broad range of ages. Importantly, this convenience sample was derived from a pre-screened, high-risk referral archive rather than a randomly selected community-based population. Consequently, the demographic distribution and high frequency of FeLV proviral DNA detected in this cohort should not be extrapolated directly to the general feline population in Thailand. The enriched clinical design aimed to increase representation of different FeLV infection profiles and facilitate comparison between molecular detection of proviral DNA and p27 antigen-based testing.

### PCR amplification

The diagnostic performance of the PCR assay targeting the FeLV *pol* gene was evaluated using 160 clinical blood specimens tested with a commercial p27 antigen rapid immunoassay. This comparative approach enabled direct assessment of molecular detection of FeLV proviral DNA against antigen-based detection within the same clinical specimens.

The *pol* gene PCR successfully amplified the expected approximately 791-bp target fragment from positive clinical specimens. As shown in Figure 2, agarose gel electrophoresis showed distinct amplicons of the expected size, corresponding to the recombinant pGEM-T::*pol* plasmid used as the positive control. The presence of clearly defined amplification products in clinical specimens demonstrated successful detection of FeLV proviral DNA under the established PCR conditions. The correspondence between the sizes of the clinical amplicons and the recombinant molecular standard further supported amplification of the intended target.

To confirm the amplified product at the sequence level, we randomly selected the amplicon from sample No. 320 for gel purification and subsequent Sanger sequencing using a primer pair adapted from Ramírez *et al.* [24]. BLAST analysis demonstrated 98% nucleotide identity with *Feline leukemia virus* isolate CHINA/HLJ/HB (GenBank accession no. MW839565) (Figure S2). This sequence identity confirmed that the PCR product obtained from the selected clinical specimen corresponded to the targeted FeLV *pol* gene sequence and provided molecular support for the specificity of the PCR assay.

### PCR assay validation

The diagnostic findings of the *pol* gene PCR and commercial p27 antigen rapid immunoassay were compared using all 160 residual EDTA-blood specimens from the enriched clinical cohort. The *pol* gene PCR detected proviral DNA in 137/160 specimens (85.6%), whereas the p27 antigen assay identified 94/160 specimens (58.8%) as positive. All 94 p27 antigen-positive specimens were also PCR-positive, and no antigen-positive/PCR-negative specimens were identified. In contrast, 43 specimens were PCR-positive but p27 antigen-negative, while 23 specimens were negative by both assays (Table 2).

**Table 2:** Comparison of FeLV pol gene PCR and commercial p27 antigen test results in an enriched Thai clinical cohort (n = 160).

***pol*** ** gene PCR**	**p27 antigen-positive**	**p27 antigen-negative**	**Total**
Positive	94	43	137
Negative	0	23	23
Total	94	66	160

Sensitivity = 68.61% (95% CI: 60.13%–76.27%); specificity = 100% (95% CI: 85.18%–100%); positive predictive value = 100% (95% CI: 96.10%–100%); negative predictive value = 34.80% (95% CI: 23.40%–47.90%); accuracy = 73.13% (95% CI: 65.55%–79.82%). CI = Confidence interval; FeLV = Feline leukemia virus; PCR = Polymerase chain reaction.

The 43 discordant PCR-positive/p27 antigen-negative specimens represented 26.9% (43/160) of the entire enriched cohort and 31.4% (43/137) of all PCR-positive specimens. Thus, nearly one-third of specimens with detectable FeLV proviral DNA were negative by p27 antigen testing in the comparative dataset. This discordance matters because it shows that detecting circulating p27 antigen and detecting FeLV proviral DNA did not identify the same subsets of cats within this enriched clinical population.

**Figure 2:**
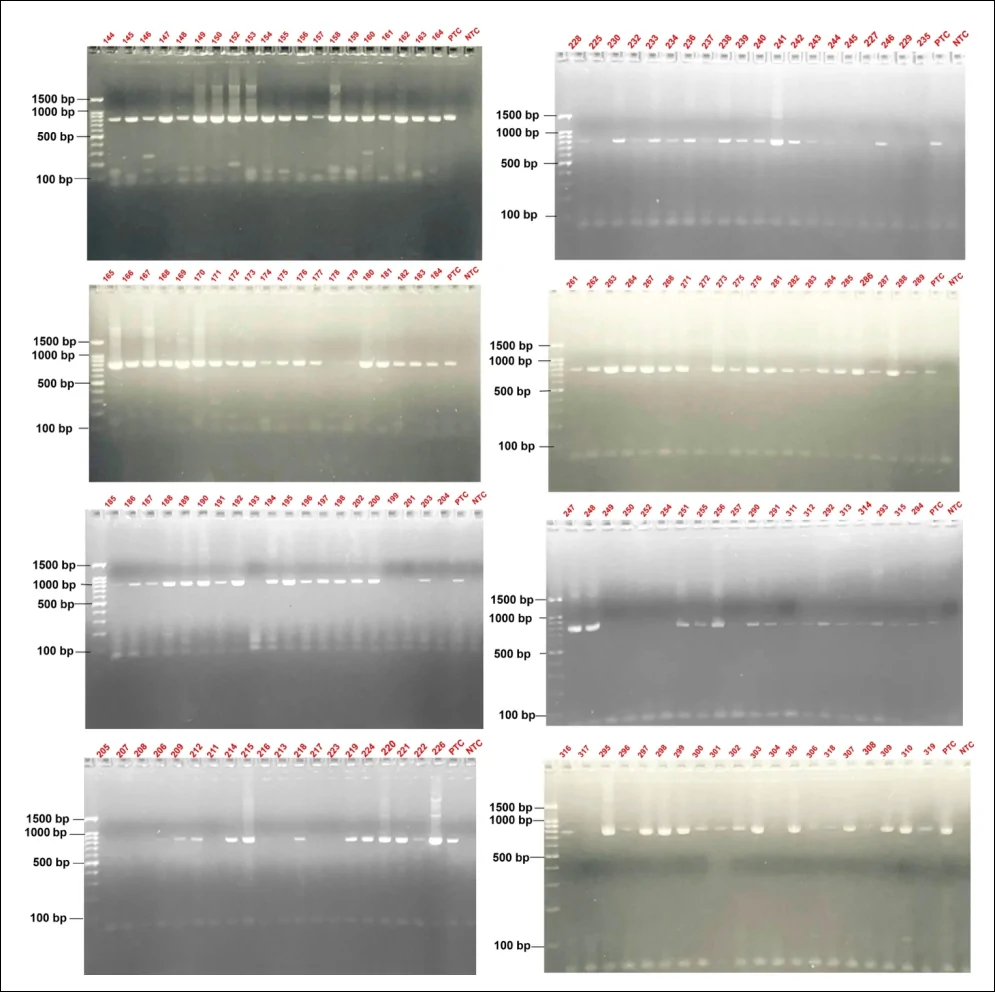
PCR amplification of the FeLV *pol* gene from clinical feline blood samples. The expected approximately 791-bp amplicon represents the FeLV *pol* gene target. FeLV = Feline leukemia virus; PCR = Polymerase chain reaction.

Using *pol* gene PCR as the reference method, the commercial p27 antigen test had a sensitivity of 68.61% (95% CI: 60.13%–76.27%) and specificity of 100% (95% CI: 85.18%–100%). The PPV was 100% (95% CI: 96.10%–100%), whereas the NPV was substantially lower at 34.80% (95% CI: 23.40%–47.90%). Overall diagnostic accuracy was 73.13% (95% CI: 65.55%–79.82%). These findings indicate that, in this enriched cohort and when PCR was the reference method, a positive antigen result agreed completely with PCR positivity, whereas a negative antigen result did not reliably exclude detectable FeLV proviral DNA.

### Molecular confirmation of discordant specimens

To further evaluate PCR-positive/p27 antigen-negative findings, selected discordant specimens were subjected to DNA sequencing. Sequence analysis confirmed that the selected PCR products corresponded to FeLV sequences (Figure S3). This molecular confirmation supports the presence of FeLV proviral DNA in the sequenced specimens despite negative p27 antigen results and provides additional evidence that the observed discordance was biological rather than attributable solely to nonspecific PCR amplification.

However, PCR-positive/p27 antigen-negative status alone should not be considered sufficient to classify every discordant cat definitively as having a latent or regressive infection. Rather, these specimens demonstrate detectable FeLV proviral DNA in the absence of detectable p27 antigen at the evaluated time point. Longitudinal antigenemia, proviral load, viral RNA, or additional confirmatory testing would strengthen classification of individual infection outcomes.

### Diagnostic performance evaluation

Receiver operating characteristic analysis was performed using *pol* gene PCR as the reference method. The commercial p27 antigen rapid test yielded an area under the curve (AUC) of 0.843 (95% CI: 0.778–0.908) (Figure 3). At its binary operating point, the antigen assay demonstrated a sensitivity of 68.61% and specificity of 100%.

Although all 94 antigen-positive specimens were PCR-positive, the p27 antigen assay did not identify 43 of the 137 PCR-positive specimens, corresponding to 31.4% of specimens with detectable proviral DNA. Accordingly, the principal diagnostic difference observed in this cohort arose from the PCR-positive/p27 antigen-negative subgroup rather than from antigen-positive/PCR-negative discordance. The AUC therefore summarizes the discriminatory performance of the binary antigen result relative to PCR status, while the underlying 2 × 2 distribution provides the clinically more informative representation of the discordance between the two detection approaches.

**Figure 3:**
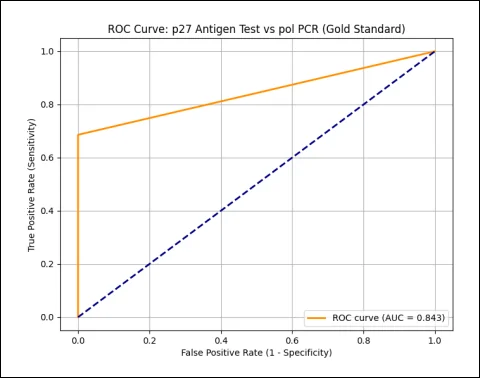
Receiver operating characteristic curve of the commercial p27 antigen rapid test compared with FeLV *pol* gene PCR as the reference method in 160 feline blood specimens from an enriched Thai clinical cohort. The area under the curve was 0.843 (95% CI: 0.778–0.908). The p27 antigen test demonstrated a sensitivity of 68.61% and specificity of 100%.

## DISCUSSION

### Diagnostic performance of *pol* gene PCR and p27 antigen testing

The principal contribution of this study is the prospective, sequence-confirmed regional validation in a Thai clinical cohort that an established *pol* gene-targeted PCR assay detects FeLV infection more comprehensively than conventional p27 antigen immunochromatography, consistent with established global evidence and extending its applicability to resource-limited Southeast Asian settings. As shown in Table 2, PCR positivity substantially exceeded p27 antigen detection, consistent with the ability of molecular testing to identify proviral DNA in both antigen-positive and antigen-negative infections. All 94 antigen-positive specimens were also PCR-positive, confirming that the molecular assay reliably detected FeLV proviral DNA in cats with concurrent p27 antigenemia. In contrast, 43 specimens were PCR-positive but antigen-negative, a diagnostic pattern consistent with regressive or latent infection, in which integrated provirus may persist despite suppression of detectable p27 expression. This discordance is consistent with the established biological spectrum of FeLV infection, whereby cats may harbor integrated proviral DNA within hematopoietic cells for prolonged periods without persistent viremia or antigenemia [5, 25].

The 23 specimens (14.4%) that were negative by both assays may reflect false-positive results during previous antigen screening or infection states in which neither p27 antigen nor detectable proviral DNA was present at the time of testing. Although abortive infection is characterized by elimination of the virus before stable proviral integration [3], the cross-sectional design of the present study does not permit definitive classification of these PCR-negative, antigen-negative cats as having resolved abortive infections. FeLV establishes persistent infection by integrating proviral DNA into the host genome, a fundamental step in the retroviral life cycle [1, 5]. Consequently, PCR detection of FeLV proviral DNA provides molecular evidence of viral genomic material even without detectable circulating antigen [12, 13]. Nevertheless, proviral DNA detection alone does not establish active viral replication or infectiousness. Thus, the 43 discordant cases are most appropriately interpreted as cats harboring detectable FeLV proviral DNA without concurrent p27 antigenemia, a profile consistent with regressive or latent infection.

The diagnostic performance estimates derived from the 2 × 2 contingency table require careful interpretation because PCR and p27 antigen immunochromatography detect different biological markers of FeLV infection. When *pol* gene PCR is considered the molecular reference method, the p27 antigen assay detected 94 of the 137 PCR-positive specimens, corresponding to a sensitivity of 68.61%, while all 23 PCR-negative specimens were antigen-negative, corresponding to a specificity of 100%. The resulting PPV was 100%, whereas the NPV was substantially lower because 43 antigen-negative specimens remained PCR-positive. This distinction is clinically important because a negative p27 antigen result did not reliably exclude detectable FeLV proviral DNA in this enriched cohort. However, predictive values are strongly influenced by infection frequency in the tested population; therefore, estimates from this deliberately enriched clinical cohort should not be extrapolated directly to the general feline population. Rather than indicating underperformance of the molecular assay, the discordance demonstrates the biological limitation of antigen testing for identifying cats in which proviral DNA persists without detectable circulating p27 protein [21, 26].

### Biological significance of PCR-positive and antigen-negative infections

The 43 PCR-positive, antigen-negative specimens are particularly relevant because their diagnostic profile is compatible with regressive FeLV infection, in which the host immune response suppresses active viral replication after initial infection while integrated proviral DNA persists. Sanger sequencing of selected discordant PCR products supported their exogenous FeLV origin. However, the absence of quantitative proviral load and transcriptional data precludes definitive determination of viral activity or replication competence in individual specimens. The documented previous p27 antigen reactivity of the source cats, if confirmed by the clinical records, would support prior productive infection; nevertheless, current PCR positivity alone cannot establish whether the detected provirus is transcriptionally active. Future studies incorporating quantitative real-time PCR for proviral copy number, preferably combined with reverse-transcription PCR for viral RNA, are required to characterize infection status more precisely and improve clinical risk stratification [12, 27].

Cats with regressive FeLV infection may retain proviral DNA for prolonged periods, and viral reactivation can occur under certain conditions of immunosuppression or concurrent disease [4, 12]. Therefore, PCR-positive, antigen-negative cats represent an important diagnostic category that may be overlooked by antigen-only screening, although their individual risk of reactivation or transmission cannot be determined from the present cross-sectional data.

### Comparison with previous molecular studies

Our finding that 43/160 cats (26.9%) were antigen-negative but proviral DNA-positive is broadly consistent with studies demonstrating discordance between antigen and molecular FeLV detection. In Brazil, Biezus *et al.* [28] used proviral DNA detection with antigen testing and identified regressive infections with clinical outcomes distinct from progressive infection, emphasizing the value of molecular testing for FeLV staging. Similarly, a study in Colombia used nested PCR targeting a conserved 306-bp region of the *pol* gene and subsequently performed *env* subtyping, demonstrating the utility of *pol*-based PCR for molecular detection and the complementary value of more variable genomic regions for molecular epidemiology [2].

In Asia, Rapichai *et al.* [14] developed a colorimetric neutral red loop-mediated isothermal amplification assay targeting the *pol* gene, providing an alternative molecular approach potentially suitable for settings with limited laboratory infrastructure. A multiplex TaqMan assay developed for cats in China likewise incorporated a conserved FeLV *pol* target and demonstrated the utility of this genomic region for molecular detection [29]. European investigations have also combined proviral DNA detection with *env* characterization to distinguish infection status and investigate viral diversity [30]. Collectively, these studies support molecular detection as a complementary approach when p27 antigen testing alone does not fully characterize FeLV infection. Nevertheless, make direct comparisons of discordance rates cautiously because estimates vary by population selection, infection stage, molecular target, assay characteristics, and sampling strategy.

Our findings also align with previous investigations showing that p27 antigen and proviral DNA assays do not always classify FeLV infection the same way. Hofmann-Lehmann *et al.* [21] demonstrated that PCR detection of proviral DNA could identify infected cats without concurrent detectable p27 antigen, particularly in the context of regressive infection. Similarly, Torres *et al.* [27] reported detection of FeLV proviral DNA among antigen-negative cats from high-risk populations. These findings, together with the 26.9% PCR-positive/antigen-negative proportion observed in the present enriched cohort, highlight a key limitation of relying exclusively on circulating p27 antigen to characterize total FeLV infection status. However, because the present population was selected based on prior clinical testing and was not randomly sampled, the observed discordance rate should not be interpreted as the expected proportion of antigen-negative/provirus-positive cats in the general Thai feline population.

### Molecular relevance of the *pol* gene target

The high degree of sequence conservation within the *pol* gene across diverse FeLV strains provides a strong molecular basis for its use as a diagnostic target. Unlike the more variable *env* gene, which is subject to genetic diversification and recombination, *pol* encodes essential viral enzymes, including protease, reverse transcriptase, and integrase, that are indispensable for the viral life cycle and consequently subject to substantial functional constraint [24, 27]. Conserving the target region reduces the likelihood of primer–template mismatches and is advantageous for molecular detection across genetically diverse viral populations. This consideration may be particularly relevant in regions such as Southeast Asia, where the genetic diversity of circulating FeLV strains remains incompletely characterized.

He *et al.* [29] similarly selected a conserved *pol* region for molecular detection in Chinese cats, whereas the greater variability of *env*, particularly among FeLV subtypes, makes that region more informative for subtype characterization and molecular epidemiology [2, 30]. In the present study, the 98% nucleotide identity observed between the sequenced clinical amplicons and reference FeLV sequences in GenBank supported amplification of the intended viral target and strengthened the molecular evidence underlying the PCR-positive findings. However, sequencing of selected products does not independently establish the analytical specificity of every PCR-positive specimen; comprehensive assay validation would require additional evaluation of analytical specificity, cross-reactivity, and the limit of detection.

### Demographic characteristics and interpretation of the enriched cohort

The demographic composition of the study cohort, which predominantly comprised young to middle-aged male Domestic Shorthair cats, is broadly consistent with demographic profiles associated with increased FeLV exposure in previous epidemiological studies. Sprißler *et al.* [18] reported associations of FeLV infection with age, sex, and behavioral factors that may increase contact with infected cats. Greater roaming activity, territorial interactions, and fighting may increase opportunities for viral exposure. Nevertheless, the present cohort was enriched through prior clinical screening and was not designed as a population-based risk-factor study. Consequently, its demographic distribution should be interpreted descriptively and should not be used to infer independent associations between sex, breed, age, and FeLV infection.

Similarly, the 85.6% PCR positivity observed in this cohort reflects the selected nature of the study population and does not represent the population prevalence of FeLV infection among cats in Thailand. The enriched sampling strategy was nevertheless appropriate for the principal objective of evaluating diagnostic discordance because it provided a substantial number of cats with detectable FeLV proviral DNA for direct comparison with p27 antigen results.

### Clinical implications for FeLV screening

Relying solely on p27 antigen immunochromatography may create a diagnostic gap in cats harboring proviral DNA without concurrent detectable antigenemia. Rapid point-of-care tests nevertheless provide important practical advantages, including rapid turnaround, relatively low cost, minimal equipment requirements, and suitability for routine veterinary practice and resource-limited settings. These advantages should be considered alongside their biological limitation: p27 assays depend on sufficient circulating viral antigen to generate a detectable signal and therefore primarily identify cats with active antigenemia [26]. When viral replication is suppressed and circulating antigen falls below the detection threshold, cats retaining proviral DNA may yield negative antigen results despite molecular evidence of infection.

Technical factors may further influence immunochromatographic assay performance, including specimen quality and volume, reagent condition, incubation time, and interpretation of weak test lines. The present study applied standardized procedures and experienced personnel, yet 43 PCR-positive specimens were antigen-negative. This finding supports the complementary use of molecular testing in selected clinical situations rather than replacement of rapid antigen testing as a practical first-line method. Previous evaluations of point-of-care p27 assays have similarly demonstrated variability in sensitivity across test platforms and study populations [20, 31, 32]. A tiered diagnostic approach may therefore be particularly appropriate for cats with discordant or previous positive results, high-risk exposure histories, clinical suspicion of FeLV-associated disease, or circumstances in which accurate assessment of proviral status is important.

The statistical parameters derived from the 2 × 2 contingency table must therefore be interpreted within the context of the biological marker selected as the reference. Using *pol* gene PCR as the molecular reference, the p27 antigen assay detected 94/137 PCR-positive specimens, yielding a sensitivity of 68.61%, whereas all 23 PCR-negative specimens were antigen-negative, yielding a specificity of 100%. The 43 PCR-positive/p27 antigen-negative specimens explain the limited ability of a negative antigen result to exclude detectable proviral DNA in this enriched population. Importantly, this finding does not imply that every PCR-positive/antigen-negative cat was actively infectious or undergoing viral replication. Rather, it shows that molecular and antigen-based assays interrogate different components of FeLV infection and that interpreting discordant results should incorporate infection biology, clinical history, and, where indicated, additional molecular or longitudinal testing.

### Predictive values and interpretation across screening populations

Within this enriched cohort, the p27 antigen test demonstrated a PPV of 100% and a NPV of 34.8% when *pol* gene PCR was used as the molecular reference. Although the observed specificity was 100%, the lower bound of its 95% CI (85.2%) indicates uncertainty around this estimate and does not exclude the possibility of false-positive results in larger or differently constituted populations. Because PPV and NPV are prevalence-dependent, their values would be expected to change substantially when the assay is applied to community, shelter, or other populations with a lower FeLV prevalence. In such settings, PPV would generally decrease and NPV increase, assuming comparable sensitivity and specificity. This prevalence dependence must therefore be considered when extrapolating the performance observed in this enriched cohort to general screening contexts.

Molecular detection may be particularly valuable when determining proviral status has important clinical consequences, including blood-donor screening, evaluation of cats with discordant or previous FeLV test results, and assessment of high-risk cats. The overall accuracy of the p27 antigen assay was 73.13%, primarily because 43 specimens containing detectable FeLV proviral DNA were antigen-negative. These findings demonstrate the additional diagnostic yield obtained by *pol* gene PCR for detecting FeLV-associated proviral DNA [13, 21]. However, the present data do not support describing PCR accuracy as approaching 100%, because a complete independent reference standard was not available and only selected PCR products underwent sequence confirmation.

### Clinical implications of proviral DNA detection

Identification of PCR-positive, antigen-negative FeLV infection has important implications for individual patient management. Such cats should not automatically be considered FeLV-free solely on the basis of a negative p27 antigen result because detectable proviral DNA indicates persistence of viral genetic material. However, PCR positivity alone does not establish active viral replication, current infectiousness, or the probability of subsequent reactivation. Clinical interpretation should therefore integrate molecular findings with antigen status, previous test results, exposure history, clinical condition, and, where available, longitudinal testing.

Cats with a PCR-positive, antigen-negative profile may warrant periodic clinical re-evaluation, particularly before interventions involving substantial immunosuppression. Blood-donor screening is especially important because transfusion recipients may be vulnerable to infectious agents, and excluding FeLV provirus-positive donors provides an additional precaution against potential transmission [6]. Nevertheless, the present study did not directly investigate transfusion transmission, reactivation rates, or transmission from PCR-positive/antigen-negative cats; therefore, management recommendations should reflect established clinical guidelines rather than assumptions based solely on the present findings.

### Feasibility of molecular testing in resource-limited settings

Implementation of *pol* gene PCR in Thai veterinary diagnostic laboratories may be feasible in facilities already equipped with conventional thermal cyclers, electrophoresis systems, and basic molecular diagnostic infrastructure. University teaching hospitals and reference laboratories are therefore potential settings for incorporating molecular confirmation into FeLV diagnostic workflows. Although conventional PCR requires laboratory equipment, trained personnel, contamination-control procedures, and additional processing time compared with point-of-care antigen testing, selective molecular testing could extend diagnostic capability without requiring PCR testing of every cat.

A tiered approach in which p27 antigen testing serves as the initial screening method and PCR is selectively applied to high-risk or diagnostically discordant cases may provide a practical balance between accessibility and diagnostic yield. Alternative amplification platforms may further increase accessibility. Rapichai *et al.* [14], for example, developed a neutral red loop-mediated isothermal amplification assay targeting the *pol* gene, illustrating the potential for equipment-light molecular detection. However, the present study did not evaluate formal cost-effectiveness. Therefore, specific claims regarding per-test costs, long-term financial savings, or the economic superiority of molecular testing require dedicated economic evaluation rather than extrapolation from diagnostic performance alone [8, 14, 33].

### Management of regressive FeLV infection

Clinical management of cats with a PCR-positive, antigen-negative profile requires careful risk stratification. Cats with regressive FeLV infection generally have a more favorable prognosis than those with progressive infection, although integrated proviral DNA may persist and reactivation can occur under particular biological or immunosuppressive conditions [28]. Quantitative assessment of proviral load, together with viral RNA detection and longitudinal antigen testing, could better differentiate infection states and provide more informative prognostic assessment than qualitative PCR alone.

Vaccination does not eliminate an established FeLV infection or integrated provirus and therefore should not be interpreted as treatment for PCR-positive cats. Preventive care against other infectious diseases should instead be individualized according to clinical status and established feline vaccination recommendations. Concurrent infections may also modify clinical outcomes. Previous evidence indicates that feline immunodeficiency virus (FIV) coinfection can influence FeLV infection dynamics and clinical prognosis [28, 34]. Thus, integrating molecular FeLV results with broader clinical and retroviral assessment may improve individualized management. The present study, however, did not evaluate longitudinal prognosis, proviral load, FIV coinfection, or treatment outcomes; these considerations should therefore be regarded as clinical implications supported by previous literature rather than direct outcomes of this investigation.

### Integration of PCR into tiered diagnostic algorithms

Integrating *pol* gene PCR into a tiered diagnostic algorithm offers a rational way to increase diagnostic yield while retaining the operational advantages of rapid p27 antigen testing. Rapid antigen testing may remain an appropriate initial screening tool because of its accessibility and short turnaround time, whereas molecular testing could be considered for cats with discordant results, previous FeLV positivity, known exposure, clinical findings compatible with FeLV-associated disease, or circumstances in which determination of proviral status is particularly important [16, 23]. Such an approach would use the two assays as complementary tools that detect different biological components of FeLV infection rather than treating them as interchangeable tests.

The cost-effectiveness of this strategy would vary among epidemiological settings. In the present enriched cohort, the antigen test had an NPV of only 34.8%, reflecting the large number of PCR-positive/antigen-negative specimens. In lower-prevalence populations, NPV would generally increase, whereas PPV would generally decrease, assuming unchanged assay sensitivity and specificity. These prevalence-dependent changes should inform decisions about the populations in which confirmatory molecular testing provides the greatest diagnostic value. Importantly, the present data do not establish a 100% NPV for PCR because no independent gold standard was available to definitively classify PCR-negative specimens. Accordingly, PCR should not be described as an infallible exclusion test on the basis of this study.

### Implications for FeLV control in high-risk populations

Failure to recognize cats harboring FeLV proviral DNA may complicate infection-control strategies in high-risk populations. The 26.9% PCR-positive/antigen-negative discordance observed in this enriched cohort demonstrates that p27 antigen testing alone did not identify all cats with detectable FeLV proviral DNA. However, because the cohort was deliberately enriched and included cats selected through previous clinical testing, this proportion should not be extrapolated directly to the broader Thai feline population.

Regressive FeLV infection remains epidemiologically relevant because proviral DNA can persist despite the absence of detectable antigenemia, and reactivation may occur under certain circumstances [4, 5, 13]. Nevertheless, the transmission potential of an individual PCR-positive/antigen-negative cat cannot be inferred solely from qualitative proviral DNA detection. Consequently, describing all such cats as active “silent reservoirs” would overstate the present evidence. Prospective studies incorporating proviral load, viral RNA, longitudinal antigen testing, and transmission outcomes are required to determine the epidemiological significance of this diagnostic category in multi-cat households, breeding facilities, and shelters.

### Practical implications for veterinary practice

The principal practical implication of improved FeLV molecular detection is the ability to clarify infection status in cats with discordant antigen results. Detecting proviral DNA can provide veterinarians with additional information to counsel owners on viral persistence, potential reactivation, follow-up testing, and clinical monitoring. In settings with limited molecular diagnostic infrastructure, establishing accessible conventional PCR or validated isothermal amplification protocols could provide a practical pathway toward more comprehensive FeLV assessment [15, 32].

However, the present diagnostic study did not evaluate population-level transmission, owner psychological outcomes, cost-effectiveness, or the effects of PCR-supported management on feline morbidity and mortality. Consequently, prospective evaluation is needed to assess the broader benefits of incorporating molecular testing into routine clinical practice. The current findings show that *pol* gene PCR identified FeLV proviral DNA in cats negative by p27 antigen testing and therefore provides complementary diagnostic information beyond antigen detection alone.

### One Health and shelter management considerations

FeLV is primarily a feline pathogen, and natural zoonotic transmission to humans has not been documented, despite experimental evidence that certain FeLV strains can replicate in some human cell systems *in vitro* [34]. Accordingly, the principal health implications of improved FeLV detection concern feline health, animal welfare, transfusion safety, and infection control rather than direct human health risk.

In shelters and other high-density feline populations, identifying cats with discordant FeLV results may support more informed management and follow-up testing [23, 35]. However, management decisions should not be based solely on a single PCR-positive result without consideration of the cat's complete diagnostic and clinical profile. The present cohort demonstrates that *pol* gene PCR can identify provirus-positive cats missed by concurrent antigen testing, but prospective operational studies are required to determine whether PCR-supported diagnostic algorithms improve specific outcomes, including transmission control, length of stay, live-release rates, and overall welfare, in Thai shelters and comparable endemic settings [35, 36].

### Study limitations

Several methodological limitations warrant acknowledgment. First, this single-center study involved an enriched hospital cohort of cats with a history of FeLV testing; therefore, selection bias may have increased the observed frequency of proviral DNA positivity and limited the generalizability of the findings to community-based or low-risk feline populations. The cross-sectional design also precluded longitudinal assessment. Consequently, we could not determine whether the 43 p27 antigen-negative/PCR-positive cats maintained persistent proviral infection, subsequently developed detectable antigenemia, experienced viral reactivation, or changed proviral status over time. This temporal limitation is important because a single timepoint cannot establish the longitudinal stability of a regressive infection or predict subsequent clinical outcomes and transmission potential.

The absence of quantitative proviral load data further limited characterization of infection status and risk stratification. Qualitative PCR demonstrated the presence of FeLV proviral DNA but could not differentiate cats according to proviral burden or establish relationships among proviral load, viral activity, clinical outcomes, and transmission potential. These constraints should be considered when interpreting the 26.9% p27 antigen-negative/PCR-positive discordance observed in this enriched cohort. Accordingly, the present findings should primarily be regarded as evidence of diagnostic discordance between antigen and proviral DNA detection and as a foundation for subsequent longitudinal and quantitative investigations.

Potential PCR inhibitors in EDTA-anticoagulated blood, including heme-associated compounds and other sample-derived inhibitors, could theoretically have affected amplification efficiency. Although stringent quality-control procedures and the high amplification success rate reduced this concern, the absence of an internal amplification control, such as a feline housekeeping gene, represents an important methodological limitation. Positive, negative, and no-template controls were included in each PCR run to monitor assay performance; however, an internal amplification control would have enabled sample-specific identification of PCR inhibition or DNA extraction failure. Future assay validation should therefore incorporate an appropriate internal control in accordance with veterinary molecular diagnostic principles [22, 23].

Formal analytical sensitivity, expressed as the limit of detection in copies per reaction or per unit volume of blood, was not established. Similarly, analytical specificity against a defined panel of related retroviruses or potentially interfering endogenous sequences and intra- and inter-assay reproducibility were not evaluated. Nevertheless, the consistent 791-bp amplicon obtained from positive clinical specimens, absence of detectable nonspecific amplification in the tested specimens and negative controls, and 98% nucleotide identity of sequenced amplicons with reference FeLV *pol* sequences provide empirical support for amplification of the intended target under the conditions used in this study. These findings, however, should not substitute for comprehensive analytical validation.

The study also did not perform parallel molecular testing targeting alternative FeLV genomic regions, such as *gag* or *env*, which could have provided complementary confirmation of proviral detection and additional information on viral genomic diversity [4, 27]. Furthermore, the single-center design at Kasetsart University Veterinary Teaching Hospital may not capture geographic variation in FeLV strains, host characteristics, exposure patterns, or feline management practices across Thailand. Multicenter investigations incorporating quantitative PCR, longitudinal monitoring, internal amplification controls, formal analytical validation, and geographically diverse feline populations would strengthen and extend the present findings [28, 32, 35].

### Practical recommendations

Based on the observed diagnostic discordance, consider incorporating *pol* gene PCR as a complementary component of FeLV diagnostic algorithms, particularly for cats with discordant or previous FeLV test results, blood-donor candidates, and p27 antigen-negative cats with substantial exposure risk or clinical findings compatible with FeLV-associated disease. Rapid p27 antigen assays remain important first-line screening tools because of their accessibility, speed, and ease of use, whereas PCR can provide additional information on proviral status when antigen testing alone does not adequately resolve infection status.

Veterinary diagnostic laboratories implementing FeLV PCR should use validated primer sets targeting conserved genomic regions together with rigorous quality-control procedures, including positive, negative, no-template, and preferably internal amplification controls. Periodic sequence confirmation may further verify target identity. Developing and validating quantitative real-time PCR assays could further improve diagnostic characterization by enabling measurement of proviral burden and longitudinal assessment of changes in proviral load. However, clinical thresholds for using proviral load to predict reactivation, transmission, or prognosis remain to be established.

### Future research

Future studies should investigate the longitudinal course of PCR-positive/p27 antigen-negative FeLV infection through prospective follow-up of affected cats. Repeated assessment using p27 antigen testing, quantitative proviral PCR, and viral RNA detection would help characterize the stability of proviral persistence, more accurately distinguish infection trajectories, and determine the frequency and circumstances of viral reactivation. Such investigations could also examine associations among proviral load, immune responses, concurrent disease, immunosuppression, clinical progression, and transmission potential.

Multicenter studies encompassing different geographic regions, clinical populations, shelters, and community cats are also required to establish population-representative estimates of FeLV infection and antigen–PCR discordance in Thailand. Cost-effectiveness analyses comparing tiered strategies, such as initial antigen screening followed by selective PCR, with broader molecular testing approaches would help determine the most appropriate use of limited diagnostic resources. Emerging molecular platforms, including loop-mediated isothermal amplification and other validated rapid nucleic acid detection technologies, may eventually facilitate molecular testing in settings without conventional PCR infrastructure. Expanded molecular epidemiological studies incorporating additional genomic targets could further characterize circulating FeLV diversity and support evidence-based disease-control strategies [6, 21].

## CONCLUSION

This study demonstrates that *pol* gene-targeted PCR provides important complementary diagnostic value to conventional p27 antigen testing for FeLV infection in an enriched Thai clinical cohort. Among 160 cats, 137 (85.6%) were positive for FeLV proviral DNA by PCR, whereas 94 (58.8%) were positive by p27 antigen testing. All 94 antigen-positive specimens were PCR-positive; importantly, 43 cats (26.9%) were PCR-positive but antigen-negative, showing that relying on p27 antigen detection alone would have missed a substantial proportion of cats harboring detectable FeLV proviral DNA in this selected high-risk population. Sequence analysis showing 98% nucleotide identity with reference FeLV *pol* sequences further supported the identity of the amplified viral target.

A major strength of this study is the paired comparison of molecular and antigen-based detection in the same clinical specimens, supported by Sanger sequencing of selected PCR products. The findings are particularly relevant to clinical situations in which accurate determination of FeLV proviral status is important. Rapid p27 antigen testing remains a valuable, accessible first-line screening method; however, selective incorporation of *pol* gene PCR may improve diagnostic resolution in cats with discordant or previous FeLV test results, high-risk exposure histories, suspected FeLV-associated disease, and blood-donor candidates. Such a tiered approach could combine the practicality of point-of-care antigen testing with the additional diagnostic information provided by molecular detection.

The findings should be interpreted within the context of the enriched, single-center study population and the absence of longitudinal testing, quantitative proviral load, and viral RNA measurements. Therefore, PCR-positive/p27 antigen-negative status should be interpreted as evidence of detectable FeLV proviral DNA rather than, by itself, proof of active viral replication, infectiousness, or inevitable reactivation. Nevertheless, identifying 43 antigen-negative cats harboring detectable proviral DNA highlights a clinically important diagnostic gap in antigen-only testing within this cohort. Overall, this study provides sequence-supported evidence for the complementary role of *pol* gene PCR in FeLV diagnosis in Thailand and supports its judicious integration into tiered diagnostic protocols to improve identification and management of cats with otherwise unrecognized proviral infection.

## DATA AVAILABILITY

The supplementary data can be made available from the corresponding author upon request.

## GENERATIVE AI DECLARATION

The authors declare that no generative artificial intelligence (AI) tools were used in the preparation, writing, editing, or revision of this manuscript.

## AUTHORS’ CONTRIBUTIONS

KU, AP, WW, NS, SC, CB, PT, JR, and AR: Conceptualization, study design, and drafted the manuscript. JR and AR: Data collection. KU, AP, WW, NS, SC, CB, and PT: Literature review. PT and AR: Data analysis and interpretation. JR and AR: Manuscript review and editing. JR: Supervision. All authors have read and approved the final version of the manuscript.
